# TNFα-blockade stabilizes local airway hyperresponsiveness during TLR-induced exacerbations in murine model of asthma

**DOI:** 10.1186/s12931-015-0292-5

**Published:** 2015-10-22

**Authors:** Magnus Starkhammar, Susanna Kumlien Georén, Sven-Erik Dahlén, Lars-Olaf Cardell, Mikael Adner

**Affiliations:** Centre for Allergy Research, Karolinska Institutet, Stockholm, Sweden; Division of ENT diseases, CLINTEC, Karolinska Institutet, Stockholm, Sweden; Institute of Environmental Medicine, Karolinska Institutet, Stockholm, Sweden; Department of ENT Diseases, Karolinska University Hospital, Stockholm, Sweden; Unit of Experimental Asthma and Allergy Research, Institute of Environmental Medicine, Scheeles väg 1, Karolinska Institutet, SE-171 77 Stockholm, Sweden

**Keywords:** Airway mechanics, Allergy, Asthma, Innate immunity, Viral infection, Bacterial infection

## Abstract

Viral infections are a common cause of asthma exacerbation. These maladies are sometimes complicated by bacterial infections. Toll-like receptors (TLRs) are in the forefront of our microbial defence, with TLR3 responding to viral and TLR4 to bacterial stimulation. The present study was designed to evaluate the effect of concomitant TLR3 and TLR4 stimulation in a murine model of allergic asthma.

BALB/c mice were stimulated intranasally with a combination of poly(I:C) and LPS activating TLR3 and TLR4, respectively. This resulted in the development of airway hyperresponsiveness (AHR) in the proximal part of the lung, along with signs of neutrophilic inflammation. Analysis of the bronchioalveolar lavage fluid (BALF) revealed a marked increase in TNFα. In contrast, the allergic airway inflammation induced by ovalbumin administration to sensitized mice caused AHR in the whole lung along with an increase in eosinophils and lymphocytes in the BALF and lung.

When poly(I:C) + LPS were given to mice with an ongoing allergic airway inflammation induced by ovalbumin, the AHR was further increased in the peripheral lung and neutrophils appeared together with eosinophils and lymphocytes in the BALF and lung. Treatment with the TNFα-blocking antibody infliximab blunted the AHR increase, without affecting the cells influx in BALF.

To conclude; a combined TLR3- and TLR4-stimulation, representing a concomitant viral and bacterial infection, causes an AHR that is further exaggerated during an ongoing allergic inflammation. The airway stabilizing effect of infliximab indicates the possible future use of TNFα blockade in treatment of microbial induced exacerbations of allergic asthma.

## Introduction

Viral infections, such as respiratory syncytial virus (RSV) and human rhinovirus (HRV) are believed to be the primary cause of asthmatic exacerbations in adults [1, 2]. *Haemophilius influenzae* and *Moraxella cattarhalis* are bacteria known to play an opportunistic role following respiratory viral infections, with their own ability to trigger exacerbations [3]. Moreover, especially *Haemophilius influenza is* frequently found together with RSV and HRV [4]. Thus, combined bacterial and viral infestations are not an uncommon problem in asthma.

Pattern recognition receptors (PRRs) is an umbrella term for several receptor families all with their specific ability to recognizing various microbes, initiating an innate host defence reaction [5, 6]. The Toll-like receptors (TLRs) are the most well characterized PRR family comprised of 10 members (13 in mice) [5]. TLR3 are known to identify viruses like RSV and HRV, whereas TLR4 recognizes bacteria like *Haemophilius influenza.* During experimental conditions poly(I:C) and LPS, ligands for TLR3 and TLR4, can be used to mimic the innate immune effects of viruses (i.e. RSV/HRV) and bacteria (i.e. *Haemophilius influenza*), respectively.

As a consequence of respiratory infections patients with allergic asthma generally suffer from prolonged and more severe symptoms than patients without established allergy [7, 8]. The mechanistic interplay between on-going allergy inflammation, microbial infections and TLR activation is complex and still poorly understood. Airway hyperresponsiveness (AHR) is closely related to the asthmatic exacerbation [2, 9]. In the present study AHR was evaluated in ovalbumin (OVA) mouse model of allergic asthma. The effect of a combined TLR3 and TLR4 activation was studied by adding poly(I:C) and LPS together once daily during four consecutive day, just after the mice had completed their sensitization protocol. The purpose was to mimic the situation when asthmatic subjects with an allergic airway inflammation are exposed to a concomitant viral and bacterial infection for a time period representing occasional infection. In addition to changes in AHR, alterations in the local respiratory tract cell and mediator composition were assessed in bronchioalveolar lavage fluid (BALF). Special attention was given to the role of TNFα which has been implicated in many aspects of airway pathology in asthma [10]. In the present study infliximab, a well-established TNFα antibody was used to investigate the involvement of TNFα in AHR.

## Methods

### Animals

Female BALB/c mice (approximately 20 g, 8–12 weeks old) were obtained from Charles River (Sulzfeld, Germany). The mice were housed in plastic cages with adsorbent bedding material in a conventional animal house with 12-hours dark/light cycles. Water and pelleted food were provided *ad libitum*. All animal procedures were approved by the regional committee of animal experimentation ethics (Stockholm norra djurförsöksetiska nämnd).

### Treatment protocol

**Fig. 1:**
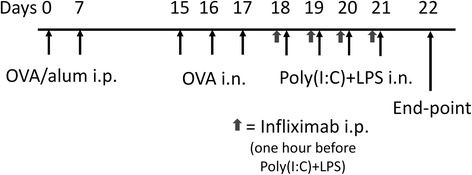
Scheme. An allergic inflammation was assessed of sensitization with 10 μg ovalbumin (OVA) and 1 mg aluminum hydroxide (alum) by intraperinoeal (i.p.) injection and thereafter challenged with 50 μg OVA intranasally (i.n.) or PBS as control. Thereafter an intranasal administration with 20 µg poly(I:C) together with 2 µg LPS (TLR3 and TLR4 agonists) or PBS as control were given during four consecutive days. In some experiments an injection of 0.1 mg infliximab i.p. were given one hour before the TLR-agonist administration

In the first set of experiments the mice were given 20 μg poly(I:C) (lyophilized; Sigma-Aldrich, St Louis, MO, USA) and 2 μg LPS (from Escherichia coli 0127:B8; Sigma-Aldrich, St Louis, MO, USA) solved in 20 μl PBS [11] or 20 μl PBS as control intranasally during isoflurane anaesthesia on four consecutive days. In the second set of experiments mice were first sensitized with 10 μg ovalbumin (OVA) (grade II; Sigma-Aldrich, St Louis, MO, U.S.A.) and 1 mg Al(OH)_3_ (Sigma-Aldrich) suspended in 200 μl PBS, given as an intraperitoneal injection (i.p.) on days 0 and 7. On days 15, 16 and 17 the animals were anaesthetized by isoflurane inhalation and 20 μl OVA (50 μg) or PBS were administered intranasally. On the subsequent four days (18–21) the animals were given poly(I:C) + LPS in the same regimen as the first set of experiments. PBS was used as vehicle control. For the second set of experiments the animals were treated with a monoclonal antibody against TNF-α (200 μl, 0.5 mg/ml infliximab (Infliximab, Centocor B.V, Leiden, The Netherlands) or PBS i.p. one hour before each Poly(I:C)/LPS administration. Lung mechanics together with collection of tissues and bronchoalveolar lavage fluid (BALF) were assessed 24 h after the last treatment (day 22) (Fig. 1).

### Lung mechanics

The mice were anaesthetised (pentobarbital sodium, 90 mg/kg bw given i.p.), put on a heating pad (body temperature, 37 °C), tracheotomised (18-gauge cannula) and connected to the *flexivent* animal ventilator (Scireq, Montreal, Canada). After ventilation was started, the mice were monitored as described earlier [11]. After a five minutes resting period, methacholine (MCh; Sigma-Aldrich) was injected through the tail vein in increasing doses (0.01, 0.03, 0.1, 0.3, 1, 3 and 10 mg/kg · body weight), in order to induce AHR.

For the first experiments, lung resistance and compliance were measured by assuming a single-compartment linear model and multiple linear regressions at a sinusoidal frequency of 2.5 Hz every eighth breath for 3 min after each injection. For the second experiment, lung mechanics were measured using a forced oscillation technique [12]. The parameters obtained were the Newtonian resistance (*R*_*N*_), a close approximation of resistance in the conducting airways; tissue resistance (*G*), which reflects energy dissipation in the lung tissue consisting of airway closure and heterogeneity of airway distribution; and tissue elastance (*H*), which characterises tissue stiffness and reflects the energy storage within the tissue.

### Bronchoalveolar lavage

Directly after lung function measurements BALF was collected (1 mL ice cold PBS containing 0.6 mM ethylendiaminetetraacetic acid (EDTA) was lavaged three times in the lung). The fluid was centrifuged at +4 °C, 1200 rpm, for 10 min and the supernatant was stored at −80 °C until use. Lysis buffer (150 mM NH_4_Cl, 10 mM KHCO_3_, 0.1 mM EDTA, pH 7.2), was used for 2 min, to lyse the red blood cells, followed by washing in PBS. Total cell number was counted and expressed as cells/ml BALF. Differential cell counts were performed on May-Grünwald/Giemsa stained cytospins, counting a minimum of 300 cells, in a blinded manner.

### Measurement of cytokines

Cytokines in BALF were measured using the Cytokine Mouse 20-Plex Panel together with RANTES Mouse Singleplex Bead Kit (Invitrogen Corp., Carlsbad, CA, USA) that were run in a Luminex200 system. The simultaneous immunoassay was carried out according to manufacturer’s instructions.

### Lung histology

Lungs were removed and immersed with 4 % buffered formaldehyde. They were then embedded in paraffin, sectioned and stained in haematoxylin and eosin. Histological findings with a focus on inflammatory cells, such as peribronchial/perivascular and parenchymal cell infiltration, were semi-quantitatively graded in a blinded manned as 0 to 4 (zero to abundant cell infiltration) and summed together. The cells were identified according to their morphology in 1000x magnification.

### Statistical analysis

Data were analysed using Graph Pad Prism®, version 5.01, software (GraphPad Software Inc., San Diego, CA, U.S.A.). Results are presented as mean ± standard error of mean (SEM) and *n* equals’ number of subjects. For comparison of airway reactivity, two-way analysis of variance (ANOVA) was followed by Bonferroni’s Multiple Comparison Test. BALF cell data and lung histology data were analysed with Kruskal-Wallis analysis of variance, followed by Dunn’s test for between group comparisons. A p value of less than 0.05 was considered significant.

## Results

### Intranasal administration of poly(I:C) + LPS for four consecutive days induces airway hyperresponsiveness with concomitant influx of inflammatory cells and release of a number of inflammatory mediators

**Fig. 2:**
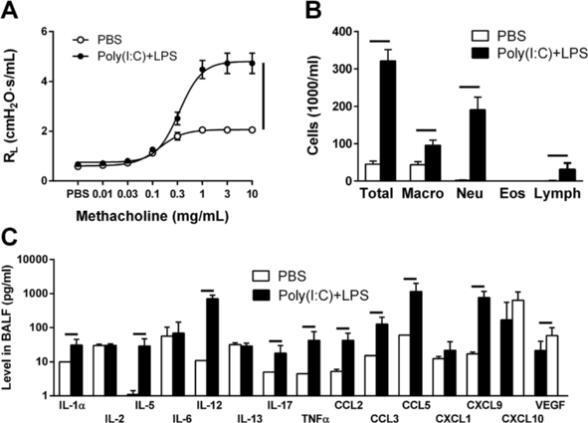
Pulmonary reactions to concomitant stimulation of TLR3 and TLR4. Responses were measured after one daily intranasal administration of 20 μg poly(I:C) + 2 μg LPS or PBS as control for four days. **a** Airways resistance after methacholine provocation. **b** Cells in BALF. **c** Cytokines measured in BALF. Results are shown as mean ± S.E.M. Lines between circles or columns represent *P* < 0.05; *n* = 10–12

To define the specific effects dual TLR activation induces on allergic airways, the effect on non-allergic airways was needed as comparison. The impact of the combined TLR3 and TLR4 stimulation on airway function was assessed by measuring the increased resistance induced by cumulative administration of methacholine. For mice given poly(I:C) + LPS, the amplitude of the lung resistance (4.88 ± 0.43 cmH_2_O^.^s^.^mL^−1^) was increased more than 2-fold when compared to the one measured in control mice (2.05 ± 0.11 cmH_2_O^.^s^.^mL^−1^) which received PBS vehicle (Fig. 2a). When evaluating the cells in BALF from the same study groups, there was a strong increase in macrophages, neutrophils and lymphocytes in the poly(I:C) + LPS treated mice compared to the control mice (Fig. 2b). No eosinophils were found in any of the groups. The BALF from these mice were processed by measuring the levels of inflammatory mediators. Ten (IL-1α, IL-5, IL-12, IL-17, TNFα, CCL2, CCL3, CCl5 CXCL9 and VEGF) out of 21 selected mediators were increased in BALF from the poly(I:C) + LPS treated mice when compared to the PBS-treated mice (Fig. 2c). For five mediators (IL-2, IL-6, IL-13, CXCL1 and CXCL10) no difference between the groups were observed and for six (IL-1β, IL-4, IL-10, IFNγ, FGF and GM-CSF) the levels were below the detection limit in both groups.

### TNFα blockade inhibits the poly(I:C) + LPS induced exaggerated airway hyperresponsiveness

TNFα, one of the mediators that was increased after the treatment with poly(I:C) + LPS, was not increased in a previous study when exposing the mice separately to the two TLR agonists with a similar protocol [11]. Thus, to investigate the role for TNFα, further experiments were performed using treatment with infliximab, an antibody against TNFα, administered i.p. one hour before the TLR-agonists were given. With this intervention, the role of TNFα was investigated both in non-allergic conditions and on an established allergic airway inflammation. In order to delineate the localization of the effect both of TLR stimulation and the TNFα action, forced oscillation techniques were used measuring the airway responses both in the conducting airways (*R*_*N*_) and in the peripheral parts, defined as tissue damping (*G*) and tissue elastance (*H*).

**Fig. 3:**
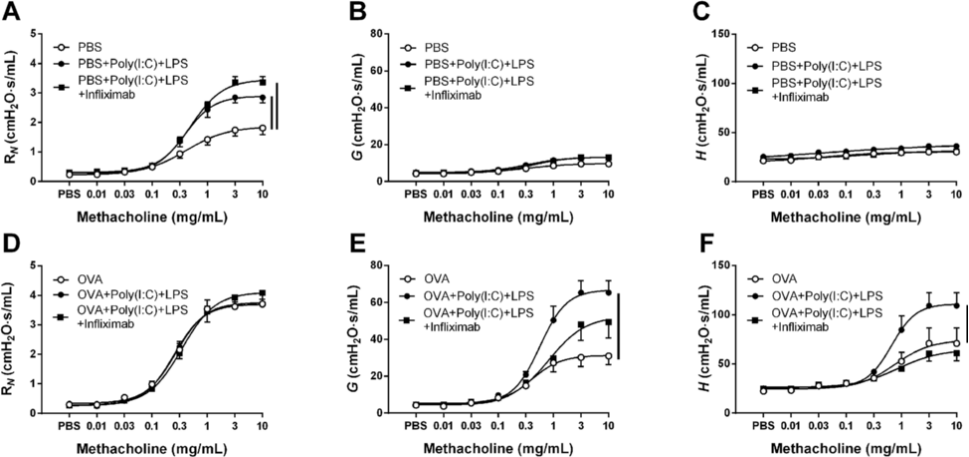
Airway responsiveness after TNFα blockade on TLR3 and TLR4 stimulation. **a** Newtonian resistance (R_*N*_), (**b**) tissue damping (*G*) and (**c**) tissue elastance (*H*) measured in PBS-treated mice (control) with one daily intranasal administration of 20 μg poly(I:C) + 2 μg LPS (P + L) for four days with and without infliximab treatment. **d** Newtonian resistance (R_*N*_), (**e**) tissue damping (*G*) and (**f**) tissue elastance (*H*) measured in OVA-sensitized and challenged mice treated with one daily intranasal administration of 20 μg poly(I:C) + 2 μg LPS for four days with and without infliximab treatment. Results are shown as mean ± S.E.M. Lines between symbols represent *P* < 0.05; *n* = 8–13

As shown when using the snap-shot technique, the treatment for four days with the combination of poly(I:C) + LPS caused AHR. However, this TLR-induced AHR was only found in the proximal region *R*_*N*_ (Fig. 3a) and not in the distal parts *G* (Fig. 3b) and *H* (Fig. 3c). Treatment with infliximab did not influence the TLR-induced increase of *R*_*N*_.

As predicted, ovalbumin sensitization and challenge caused an AHR in both the proximal (Fig. 3d) and distal parts of the lung (Fig. 3e-f) [12]. When poly(I:C) + LPS was administered after an allergic airway inflammation was established, the treatment did not cause a further increase in AHR in *R*_*N*_. In contrast, the TLR treatment caused a marked increase in AHR in both the distal parameters *G* and *H*. This increased effect of the TLRs on *G* and *H* on the established allergic airway inflammation was markedly inhibited by infliximab whereas the infliximab treatment did not affect the *R*_*N*_.

### TNFα blockade does not inhibit the poly(I:C) + LPS induced exaggerated cellular influx in bronchoalveolar lavage fluid

**Fig. 4:**
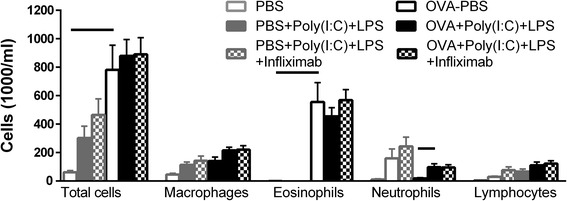
Cellular composition in BALF after TNFα blockade on TLR3 and TLR4 stimulation. BALF was collected from control or OVA-sensitized and challenged mice treated with one daily intranasal administration of 20 μg poly(I:C) and 2 μg LPS for four days with and without infliximab treatment. Results are shown as mean ± S.E.M. Lines between columns represent *P* < 0.05; *n* = 8–13

As described in Fig. 2, administration of poly(I:C) + LPS to non-sensitized mice again increased the neutrophils, macrophages and lymphocytes. This TLR-induced increase was not affected by concomitant infliximab treatment (Fig. 4). OVA-exposed mice showed a marked increase in eosinophils. Poly(I:C) + LPS administration to the mice with the established allergic inflammation did not influence the eosinophils, but caused an increase in neutrophils and a trend to increasing both macrophages and lymphocytes. This augmentation by TLR-stimulation was not affected by the infliximab treatment.

### TNFα blockade does not affect the poly(I:C) + LPS induced increase in inflammatory cell infiltration in lung tissue

**Fig. 5:**
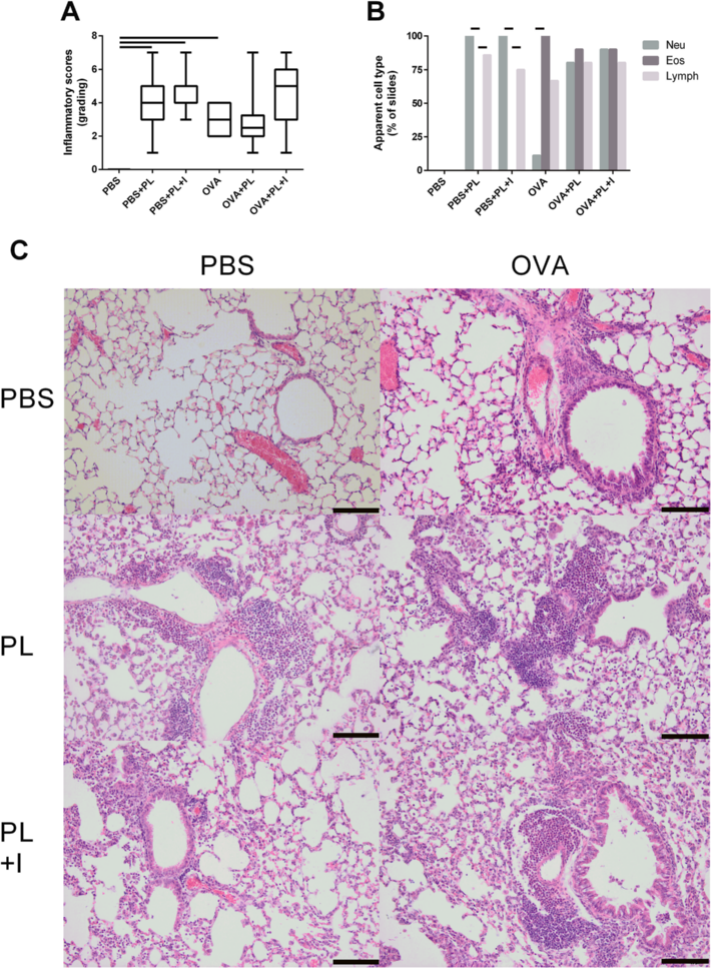
Cellular infiltration in the lung after TNFα blockade on TLR3 and TLR4 stimulation. Lungs was collected from control or OVA-sensitized and challenged mice treated with one daily intranasal administration of 20 μg poly(I:C) and 2 μg LPS (PL) for four days In absence and presence of infliximab (+I) treatment. **a** Compiled data of semi-quantitatively graded cellular infiltration shown as box plot (*n* = 6–10). **b** Identification of the cell types that were apparent in the slides shown as percentage (*n* = 6–10). Lines between columns represent *P* < 0.05. **c** Typical pictures of histological sections stained in haematoxylin and eosin (200 x magnification; lines respresent 100 μm)

Administration of poly(I:C) + LPS to non-sensitized mice also caused a strong influx of cells in the lung which not was altered by infliximab-treatment (Fig. 5a, b). The influx consisted of neutrophils and lymphocytes (Fig. 5c). A strong influx of cells in the lung tissue was also seen in OVA-exposed mice (Fig. 5a, b). The main cell types were eosinophils and lymphocytes (Fig. 5c). In the lung from mice with the established allergic inflammation and exposed to Poly(I:C) + LPS a similar strong influx of cells was shown although with additional neutrophils (Fig. 5a-c) This influx was not affected by the infliximab treatment.

## Discussion

The present study showed that intranasal installation of poly(I:C) together with LPS for four days in mice triggered an AHR and airway inflammation with increased influx of macrophages, neutrophils and lymphocytes, and enhanced release of several pro-inflammatory cytokines including TNFα in BALF. These TLR-induced effects were not affected by treatment with the anti-TNF antibody infliximab. In mice with established allergic airway inflammation, characterized by an AHR and an eosinophilic influx in BALF, the administration of poly(I:C) + LPS caused a further increase of AHR in the peripheral lung; tissue damping and tissue elastance. This TLR agonist-induced increase of AHR was blocked by infliximab, but it did not inhibit the additional influx of macrophages, neutrophils and lymphocytes in BALF. Thus, the present study indicates that TNFα causes an AHR in the peripheral lung in allergic inflammatory airways which not is linked to cellular influx in BALF.

Four days intranasal administration of poly(I:C) together with LPS, mimicking an effect of a combined viral and bacterial stimulation during a time period representing occasional infection, caused a marked AHR in mice. In our earlier studies, where these two TLR agonists were given separately in a similar regimen, both of them induced AHR [11, 13]. However, the mean maximal effect on the lung resistance (*R*_*L*_) was clearly higher for the combination of poly(I:C) together with LPS in this study (4.9 cmH_2_O^.^s/ml) compared to the effect when either of the TLR agonist were given alone which did not exceed 3.5 cmH_2_O^.^s/ml. Isolated mouse airways, which express both TLR3 and TLR4 in the smooth muscle layer, become hyperreactive when exposed to either poly(I:C) or LPS [14]. In the same study it was shown that when the two TLR agonists were given together this caused a synergistic increase of the hyperreactivity [14]. Thus, it is possible that the combined effect of intranasal challenge with poly(I:C) or LPS cause is due to a direct action of TLR3 and TLR4 on airway smooth muscle layer which causes AHR.

The combined treatment with poly(I:C) and LPS also caused an inflammation shown both by the influx of cells in BALF and lung tissue as well as by the recorded release of inflammatory mediators in the airways. Macrophages, neutrophils and lymphocytes were found to be increased in BALF. As it has previously been shown that poly(I:C) induces lymphocytes and LPS induces neutrophils [11, 13], the increase of both neutrophils and lymphocytes in BALF in the present study suggests additive effects for the TLR agonists. The infiltration in lung was also dominated by neutrophils and lymphocytes. As an additional marked increase in macrophages was found in BALF, this indicates that the response is more than simply summation of effects. When analyzing the release of inflammatory mediators, the same pattern as for the cells appears. Hence, those mediators that were previously found to be increased by poly(I:C) (IL-5, IL-12, CCL2 and CXCL1) and by LPS (CXCL9 and VEGF) [11, 13] were also, with the exception of CXCL1, increased by the combined TLR agonist treatment. In addition, the levels of IL-1α, IL-17, TNFα, CCL3 and CCL5 were only increased when poly(I:C) and LPS were given together. This synergistic effect of poly(I:C) + LPS in causing TNFα secretion has previously been shown in macrophages and osteoclast precursors [15, 16]. As TNFα release is a supposed to be an early event and it can stimulate the induction of various inflammatory genes [17], it is possible that several of the other mediators are induced due to the effect of TNFα. Thus, it appears that the TLR agonists both induce an effect by themselves and their combined activation causes an interaction that generates further effects.

Although clinical studies using antibodies against TNFα to treat asthma has shown variable [18], the initial clinical studies using TNFα-antibodies showed improved effect specifically on AHR [10, 19]. Interestingly, a subgroup of severe asthmatic patients also suffering from rhinosinuitis responded very well to anti-TNFα treatment [20]. To evaluate the importance of the TLR-induced effect by TNFα on the development of AHR in this study, the mice were treated with the TNFα antibody infliximab during the four days of intranasal administration of poly(I:C) + LPS. In these experiments a forced oscillation technique was used to localize which parts of the airway were affected. As in the first experiment, the combination of the TLR agonists induced AHR. However this was found to be localized in the conducting airways (*R*_*N*_) and not in the peripheral tissue damping (*G*) and tissue elastance (*H*). Previous experiments also investigating the pulmonary resistance using a forced oscillation technique showed the same pattern for the TLR9-agonist CpG oligodeoxy-nucleotide 2006 which only caused an increase of *R*_*N*_ with no effect on *G* and *H* [21]. As the conductive airways is the part of the lung that contain smooth muscle cells it further strengthen the suggestion above that the TLR-agonists given in non-allergic healthy conditions cause a hypercontractile smooth muscle a poly(I:C) + LPS [14, 22] which is responsible for the AHR. However, the effects by the dual TLR stimulation on *R*_*N*_, the cellular influx in BALF and infiltration of cells in the lung were not affected by infliximab indicating that TNFα has only a minor effect on smooth muscle and cellular trafficking in this model.

The sensitization and challenge of OVA also induced an AHR which at *R*_*N*_ was similar to that induced by the administration of poly(I:C) + LPS but it caused a higher AHR in both the peripheral parameters. This level of AHR was observed five days after the last OVA challenge, indicating a persistent allergic airway inflammation as described previously [13, 21]. When poly(I:C) + LPS were given for four days on an established allergic inflammation, the AHR was further increased as measured by both the peripheral parameters *G* and *H* but the TLR-agonists did not affect the conducting airways (*R*_*N*_). An increase in lung resistance has been shown in response to both poly(I:C) and LPS when they were given separately on a similar allergic airway inflammation [13]. However comparison of the level of response is not possible as the effect in different compartments of the lung was not determined in that study. It has been shown that when *G* and *H* increase in a similar manner it depends on airway closure [23] which is a major component of hyperresponsiveness in allergically inflamed airways [24]. One important factor for airway closure is the production of surfactant which has shown to be decreased by both viral [25] and TLR4 activation [26]. Thus, the increase of the combined response to TLR3 and TLR4 suggests that there may be a specific effect of combined viral and bacterial infection on peripheral dysfunction of the lung during allergic conditions. Considering that there is a substantial amount of evidence that inflammation of the small airways contributes to the clinical expression of asthma [27], the markedly difference of AHR caused by TLR activation during allergic (peripheral) and non-allergic (central) may be one reason for the severity for asthmatic subjects during infections.

When infliximab was given on the established allergic inflammation one hour before the TLR-agonist administration the increased peripheral AHR was markedly reduced, without any effect on the influx of cells in the BALF or the infiltration of cells in lung. These results indicate that TNFα has a specific effect on the peripheral AHR, which has been suggested to be dependent on heterogeneous airway closure due to protein interference with surfactant [28]. Through the separation of the lung mechanics it could be shown that the response on *R*_*N*_ of TLR-stimulation on non-provoked airways differed from the responses when the TLR-agonists were given on an established allergic airway inflammation. The reason may be that the latter condition induces a resistance close to the maximal capacity of this parameter. On the other hand, that the TLR-agonist-induced increase of AHR in the peripheral parameters was caused by TNFα may be of specific importance since airway closure is linked to excessive bronchoconstriction [29], recurrent exacerbations [30] and asthma severity [31]. It is possible that several other inflammatory mediators also are reduced secondary to the reduction of TNFα. The interpretation is further complicated by the effects TNFα by itself has on the airways [32]. It is also important to keep in mind that secondary stimulus like IL-1β [33] can be directly linked to TNFα.

To summarize, a combined TLR3- and TLR4-stimulation, representing a concomitant viral and bacterial infection, caused an AHR that was further exaggerated during an on-going allergic inflammation. It is likely that there is a synergistic interplay between the two innate receptor subtypes that contributes to the worsening of the situation for the mice as reflected in an increased AHR and elevated levels of a multitude of BALF mediators. It is interesting to notice that the TLR-induced increase of AHR in our model was dampened when the mice were treated with infliximab suggesting a pivot role for TNFα in microbial induced exacerbation of allergic asthma. The outcome of previous attempts to use various TNFα blocking agents in asthma has been meagre [18]. Most of these drug evolutions have been dealing with asthma from a more general perspective, and it could be that the role of TNFα is of specific importance only during the circumstances reflected in the present set up. It should be stated that the experimental set-up in this study was performed to define the action of TNF during infection and not to investigate the effect as add-on therapy for asthmatic subjects exposed to infections. Hence, further studies needs to outline whether it might be a future for specific anti-TNFα trials focusing on exacerbations of allergic asthma.
